# Potential Activity of 6-Pentyl-α-pyrone as an Antiviral for Bovine Coronavirus

**DOI:** 10.3390/pathogens15030332

**Published:** 2026-03-20

**Authors:** Luca Del Sorbo, Rosa Giugliano, Clementina Acconcia, Maria Michela Salvatore, Alessia Staropoli, Violetta Iris Vasinioti, Maria Stella Lucente, Paolo Capozza, Francesco Vinale, Annamaria Pratelli, Luigi Russo, Rosa Iacovino, Filomena Fiorito

**Affiliations:** 1 Department of Veterinary Medicine and Animal Production, University of Naples Federico II, 80137 Naples, Italy; luca.delsorbo2@studenti.unina.it (L.D.S.); rosa.giugliano@unicampania.it (R.G.); mariamichela.salvatore@unina.it (M.M.S.); francesco.vinale@unina.it (F.V.); 2 Department of Environmental, Biological and Pharmaceutical Sciences and Technologies, University of Campania Luigi Vanvitelli, 81100 Caserta, Italy; clementina.acconcia@unicampania.it (C.A.); luigi.russo2@unicampania.it (L.R.); 3 Department of Agricultural Sciences, University of Naples Federico II, 80137 Naples, Italy; alessia.staropoli@unina.it; 4 Institute for Sustainable Plant Protection, National Research Council, 80055 Naples, Italy; 5 Department of Veterinary Medicine, University of Bari, 70010 Bari, Italy; violetta.vasinioti@uniba.it (V.I.V.); mariastella.lucente@uniba.it (M.S.L.); paolo.capozza@uniba.it (P.C.); annamaria.pratelli@uniba.it (A.P.)

**Keywords:** BCoV, 6PP, AhR, MDBK, antiviral activity, in vitro, in silico

## Abstract

During infection in vitro with the strain 438/06 of bovine coronavirus (BCoV), a β-coronavirus similar to severe acute respiratory syndrome (SARS) CoV-2, treatment with 6-pentyl-α-pyrone (6PP), a fungal metabolite obtained from *Trichoderma atroviride*, was recently shown to influence viral load by reducing viral entry. Herein, the ability of 6PP to counteract the BCoV infection was further investigated both in vitro and in silico. Following the BCoV (strain 282/23) infection in bovine (MDBK) cells, the 6PP in co-treatment increased cell viability, reduced morphological signs of cell death, and significantly inhibited viral yield, by lessening the expression of the viral spike (S) protein, as well as the gene transcription of the viral nucleocapsid (NP) protein. In addition, a noticeable down-regulation in the expression of aryl hydrocarbon receptor (AhR) signaling, a strategic modulator of CoVs infection, was found. Molecular docking studies were performed to evaluate the potential interaction between 6PP and AhR involved in the BCoV infection. The docking 3D structural model showed that 6PP fits into a binding pocket positioned between the PASB and TAD domains of bovine AhR (bAhR), where the ligand is stabilized through hydrophobic interactions. In addition, the obtained computational data strongly suggest that the bAhR binding mechanism of 6PP is principally mediated by a well-conserved hydrophobic cavity playing a key role in the modulation of the receptor functions. Overall, our findings showed an antiviral action of 6PP versus BCoV infection in vitro and in silico.

## 1. Introduction

The rapid spread and high frequency of genomic mutations in coronaviruses (CoVs) may eventually escape the antiviral effect of the known antiviral compounds, and that makes the identification of new molecules with antiviral activity necessary. BCoV shares some common features with SARS-CoV-2—they are both β-CoVs, having a wide host range and a tropism for the respiratory and gastrointestinal system [1]. To date, FDA has approved only a few molecules for the treatment of the SARS-CoV-2 infection, among which nirmatrelvir and ritonavir are used as a combined therapy in adults, remdesivir for selected adult and child patients, and baricitinib and tocilizumab for selected adults under hospital care [2]. However, side effects could be provoked by these drugs [2], which generally act as classical antivirals on targets implicated in viral replication [3,4,5,6,7]. These events make the continuous search for antiviral compounds against SARS-CoV-2 necessary, often by new mechanisms of action. In this perspective, the aryl hydrocarbon receptor (AhR)—a gene regulator controlled by substances generated inside an organism comprising biliverdin and bilirubin as well as external compounds like pollutants, some drugs, and microbial products—also regulates the immune system during viral infections [8,9,10]. Consequently, AhR is modulated by several physiological functions, including embryonic as well as adult tissue development, energy metabolism, chemical and microbial protection [8,9,10]. The canonical pathway shows that, following ligand binding, the cytoplasmic AhR–ligand complex is involved by nuclear translocation due to AhR Nuclear Translocator (ARNT), then it interacts with specific DNA regions to regulate gene expression of cytochrome P450 biological catalysts (including CYP1A1, CYP1B1, and CYP2A1). This process leads to cytokine secretion and modulation of immune responses [9,10]. Moreover, AhR activation triggers the production of AhR Repressor, that then competes with AhR for ARNT to block new gene transcription. After that, the complex ligand-AhR is shifted to the cytoplasm for degradation by 26S proteasome, keeping the system in balance [9]. In infection, AhR inhibits innate immune defenses against several microorganisms [9,10,11,12,13]. Indeed, RNA viral infections due to Zika virus, influenza A virus, and CoVs provoke AhR up-regulation, which alters cytokine levels, determining variations in the immune response of host and then causing progression in viral replication [8,9]. Particularly, AhR is activated by infections provoked not only by α-CoVs, like human coronavirus (HCoV)-229E, feline coronavirus, canine coronavirus (CCoV) and porcine epidemic diarrhea virus, but also by β-CoVs, including SARS-CoV-1, SARS-CoV-2, mouse hepatitis virus (MHV), HCoV-OC43, Middle East respiratory syndrome-CoV, and BCoV [12,14,15,16,17,18,19,20,21], as well as γ-CoVs like the avian infectious bronchitis virus [22].

Specifically, following MHV infection, a decrease in the levels of cytokines interleukin (IL)-1b and IL-10 and an increase in the level of the tumor necrosis factor were provoked by using CH223191, the selective antagonist of AhR [9]. Interestingly, an opposite trend was induced by dioxin, the specific AhR agonist [9]. In addition, both CH223191 as well as 3′,4′-dimethoxy-α-naphthoflavone, another AhR-inhibitor, interfere with the replication of HCoV-229E, SARS-CoV-2 and CCoV [15,16,17,18]. Remarkably, targeting AhR during CoV infection [18,20,21,23,24] highlight the potential role of AhR in fighting against CoV infection. Surprisingly, with regard to SARS-CoV-2, pharmacological inhibition of AhR blocks SARS-CoV-2 replication [15,16,17]. Several drugs originate from various natural products including naturally occurring compounds or compounds obtained by them [25]. Fungi are widely studied for their ability to produce bioactive compounds that find application in various fields, like medicine and veterinary medicine. In fact, SMs are known for their valuable activity such as antibiotic, antifungal, and herbicidal, as well as antivirals [23,26,27,28,29,30].

Among SMs, 6-pentyl-α-pyrone (6PP) has revealed different activities, i.e., against plant pathogens [31,32] as well as in anti-biofilm-producing bacteria [33]. In vitro anti-CoV properties have been recently shown by 6PP, reducing CCoV infection [23], and lowering viral load by a modulation of BCoV (strain 438/06) entry [34]. This metabolite belongs to the family of 2-pyrones with a motif characterized by six-membered cyclic unsaturated ester frequently produced by bacteria, fungi, plants and insects [32,35]. Interestingly, it has been shown that 6PP, as well as other fungal SMs, exert their anti-CoV activity by involving AhR [23,26,36,37].

It has also been established that β-CoVs utilize lysosomes as an entry mechanism into the host cell, leading to lysosomal deacidification, despite their typically acidic environment [38]. Interestingly, natural substances, like fungal SMs, which have potential antiviral activity against CCoV [39] and BCoV [37], can also determine cellular stress on lysosomes’ pH. Although BCoV entry in the host cells did not require an acidic environment [40], following BCoV infection, a significant alkalinization of lysosomes was observed [37]. Hence, herein, following BCoV infection in vitro, we have evaluated the effect of 6PP on the pH of lysosomes. In addition, AhR signaling was assessed during infection and treatment with 6PP.

**Figure 1:**
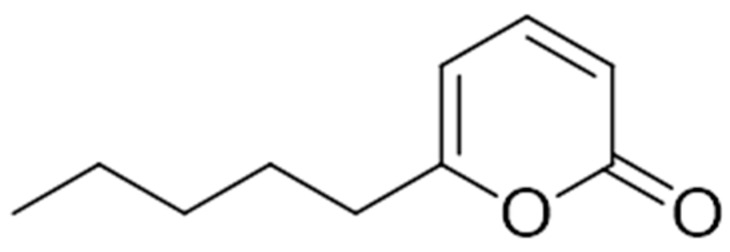
Chemical structure of 6-pentyl-α-pyrone (6PP).

In this study, after isolation of 6PP from the fungus *Trichoderma atroviride* P1 (Figure 1), its potential activity as an antiviral to fight against the BCoV (strain 282/23) infection has been investigated in Madin–Darby Bovine Kidney (MDBK) cells. In this context, several field strains of BCoV grow weakly in cell culture and are unsuccessful in developing cytopathic effects (CPEs), but cell culture-adapted strains of BCoV replicate in a lot of bovine cells, including embryonic lungs, bovine skin cells, and MDBK. Thus, MDBK cells represent a good cell–substrate for studying BCoV, as previously reported [20,34,37,41,42,43,44,45].

Herein, the investigations were performed after evaluating the non-toxic in vitro concentrations of 6PP by using this natural compound in co-treatment during BCoV infection in MDBK cells. Different experimental conditions (e.g., temperature, pre- and post-infection, and time of incubation) compared to those previously reported in Vasinioti et al., 2025 [34] were followed by us.

For the first screening phases in the identification of natural active compounds useful to fight CoVs, BCoV is an example of a good option for performing initial tests of the power of new potential drugs, avoiding the hazards due to the manipulation of highly pathogenic viruses. Furthermore, molecular docking research was performed to assess the potential interaction between 6PP and AhR in BCoV infection.

## 2. Materials and Methods

### 2.1. Production, Isolation and Identification of 6PP

6-Pentyl-α-pyrone (6PP) was extracted from the strain P1 of *Trichoderma atroviridae* following a procedure adapted from Vinale et al. [31]. In short, five 10 mm diameter agar plugs, taken from lively growing P1 cultures, were used to inoculate two 5 L Erlenmeyer flasks, each including 2.5 L of potato dextrose broth (PDB, HI-MEDIA, Pvt. Ltd., Mumbai, India). Then, incubation of the static cultures was carried out at 25 °C for 30 days before being vacuum filtered through Monodur filter paper.

The resulting 5 L of culture filtrates was subjected to complete extraction with ethyl acetate (EtOAc, VWR International, LLC, Milan, Italy). The combined organic extracts were then dried over anhydrous sodium sulphate (Na_2_SO_4_, VWR International) and concentrated by rotary evaporation under vacuum at 37 °C. The subsequent purification of 6PP was performed via flash column chromatography, employing silica gel (100 g) as the stationary phase and a gradient elution system of petroleum ether (Carlo Erba, Milan, Italy) and EtOAc (0–100% EtOAc). Characterization of the purified 6PP was performed using gas chromatography–mass spectrometry (GC-MS, Agilent 5977B MSD mass spectrometer coupled to an Agilent 8890 GC, Agilent Technologies, Santa Clara, CA, USA) as described by Staropoli et al. [46] (Figure S1). A 6PP standard (Sigma-Aldrich, St. Louis, MO, USA) was employed for quantification purposes [43].

### 2.2. Cell Cultures and 6PP

MDBK (ATCC, CCL-22) cells were cultured in Dulbecco’s modified Eagle’s minimal essential medium (DMEM) with supplement of 10% fetal bovine serum (FBS) [45].

6PP was dissolved in dimethyl sulfoxide (DMSO) (Sigma-Aldrich, St. Louis, MO, USA) to obtain a dose of 0.1 g/mL (stock solution) and then added to the medium to have the no-cytotoxic dose of 0.1 µg/mL (corresponding to 0.6 μM), as previously reported [34]. DMSO in DMEM (0.1% *v*/*v*) was used as a vehicle control.

### 2.3. Virus Production

BCoV, strain 282/23, collected by the Sector of Infectious Diseases in the Department of Veterinary Medicine (at the University of Bari Aldo Moro in Valenzano, Italy), was cultured and titrated in MDBK cells [20,37].

### 2.4. Cytotoxicity Time-Course

The determination of the cytotoxicity time-course of 6PP (0.1 µg/mL) in MDBK cells was performed by using the Cell Proliferation Kit I 3-(4,5-dimethyl-2-thiazolyl)-2,5-diphenyl-2H-tetrazolium bromide (MTT) (Roche, Basel, Switzerland) assay, as previously reported [20,36,47]. The MTT test was used to detect changes in the mitochondrial dehydrogenase activity in 6PP-treated cells compared to control cells.

To examine the cytotoxicity time-course, cells were seeded in 96-well plates (5 × 10^3^ cells/well), and incubated at 37 °C. After 24 h, cells monolayers were treated with 6PP (0.1, µg/mL) and incubated for 72 h. After that, cell viability was assessed by MTT assay, by adding 10 µL of MTT solution (5 mg/mL) to each well, as described in the manual, and by measuring the absorbance at 540–560 nm (A540/560) by Thermo Scientific™ Multiskan™ FC Microplate Photometer (Thermo Fisher Scientific, Waltham, MA, USA). The number of living cells over the total cell number was determined as the percentage and the results are reported as the mean ± S.D. of three independent experiments.

### 2.5. Antiviral Activity

MDBK cells were seeded in 96-well plates and infected with BCoV strain 282/23, at multiplicity of infection (MOI) of 0.05 or 0.5, and treated with DMEM supplemented with 10% FBS containing 6PP (0.1 µg/mL) to have four groups: (a) untreated uninfected cells; (b) untreated infected cells; (c) 6PP-treated uninfected cells; and (d) 6PP-treated infected cells. After 1 h of adsorption at 37 °C, cells were incubated and processed at 72 h post-infection (p.i.). Moreover, to investigate the involvement of AhR signaling at intermediate times, by using different MOI, we also processed cells at 48 h p.i. In each antiviral assay, BCoV was in the culture medium for the entire experiment.

#### 2.5.1. Viral Inhibition Assay

The determination of cell viability during infection was performed by the MTT assay. Monolayers of MDBK cells were either infected or not with BCoV at MOI of 0.05, in the presence or in the absence of 6PP (0.1 µg/mL). At 72 h p.i., the MTT assay was performed as above.

Additionally, following infection with BCoV at a MOI of 0.05 for 72 h, viral CPE inhibition was evaluated in infected cells treated or not with 6PP by microscopy, using a ZOE Fluorescent Cell Imager (Bio-Rad Laboratories). Due to viral replication in infected cells, cytopathogenic viruses provoke CPE. Indeed, criteria for CPE development are characterized by cellular lysis and development of areas of morphological changes like the destruction of cellular sheet, presence of giant cells, and syncytia [18].

#### 2.5.2. Viral Titer Inhibition Assay (Reed and Muench)

MDBK cells, treated with 6PP (0.1 µg/mL) or untreated, were infected or mock infected with BCoV at a MOI of 0.05, incubated at 37 °C, and handled at 72 h p.i. Tissue infectious dose 50 value (TCID_50_) of 1 × 10^9.16^/mL of BCoV was used to infect the cells. After 1 h of adsorption at 37 °C, cells were incubated and processed 72 h post p.i. BCoV was in the culture medium for the entire experiment. Viral titration was performed using the TCID_50_ method, in line with Reed and Muench (1938), as described [37].

### 2.6. Examination of Cell Morphology and Cell Cytoskeleton

Cell monolayers were infected or not with BCoV (MOI 0.05) in the presence or absence of 6PP (0.1 µg/mL) and incubated for 72 h. Subsequently, cells were washed with PBS, stained with Giemsa, acridine orange/propidium iodide (AO/PI) [23,48,49] and Phalloidin for the detection of morphological signs of cell death [50,51,52].

Giemsa staining was carried out on fixing (95% ethanol), draining and drying cells, which were then stained with a 5% Giemsa solution (Merck, Darmstadt, Germany) for 30 min. After that, the cells were rinsed (tap water and H_2_O), and checked via light microscopy (ZOE Cell Imager, Bio-Rad Laboratories, Hercules, CA, USA).

AO, the remaining membrane-permeable, combines nucleic acids and emits green fluorescence. In contrast, PI, which cannot penetrate intact cell membranes, enters only dead or dying cells, where it intercalates with nucleic acids to form a bright red, fluorescent complex. The excitation/emission wavelengths used were 460 nm/650 nm for AO, and 535 nm/617 nm for PI.

Actin filaments of cell cytoskeleton were stained by fluorescent Phalloidin solution through incubation of cells with Phalloidin-Atto 488 (Sigma-Aldrich, Milan, Italy), as previously described [53] and observed under the fluorescence microscope ZOE Fluorescent Cell Imager (Bio-Rad Laboratories). To corroborate that the effect on the cytoskeleton of infected cell was due to BCoV, we performed the assay by using a specific viral marker like mouse anti-Bovine Coronavirus Spike (S) Antibody (5A4) (MAB12430, The Native Antigen Company, Oxford, UK) (1:400) and goat anti-mouse Alexa Fluor 546 (Thermo Fisher Scientific) (1:500).

The quantification of fluorescence signals was performed by ImageJ software (version Java 1.8.0_3454; National Institutes of Health, Bethesda, MD, USA) on a Windows 10 operating system. TIFF-formatted images were imported into the software, and measurements were obtained by selecting the “Measure” function within the “Analyze” menu, which yields multiple quantitative parameters, hence, the integrated density, defined as the sum of pixel intensity values, was obtained. The fluorescence intensity of the specific proteins was quantified in 20 fields/image. Statistical analysis was carried out on results from 3 images/group. Plot bars were the mean ratio generated from the integrated density (product of the area and mean fluorescence intensity) [20,21,24,37].

### 2.7. Immunofluorescence

MDBK cells, in a 96-well plate, were treated or not with 6PP (0.1 µg/mL) and infected or not with BCoV at MOI 0.05 or 0.5 for 48 h. After that, immunofluorescence staining was carried out [36,54]. The following antibodies and antisera were used after dilution in 5% bovine serum albumin-1x Tris-Buffered Saline, 0.1% Tween^®^ 20 Detergent: (a) anti-aryl hydrocarbon receptor (AhR) (Sigma-Aldrich, St. Louis, MO, USA) (1:250); (b) mouse anti-Bovine Coronavirus Spike Antibody (5A4) (MAB12430, The Native Antigen Company, Oxford, UK) (1:400); (c) mouse monoclonal anti-CYP1A1 (A-9) (sc-393979, Santa Cruz Biotechnology, Inc., Santa Cruz, CA, USA); (d) goat anti-rabbit Texas Red (Thermo Fisher Scientific) (1:500); and (e) goat anti-mouse Alexa Fluor 488 (Thermo Fisher Scientific) (1:500).

The fluorescence intensity of the specific proteins from microscopy images, assessed by ZOE Fluorescent Cell Imager (Bio-Rad Laboratories), was determined by ImageJ, as above. Statistical analysis was carried out on results from 3 images/group. Plot bars were the mean ratio generated from the integrated density (product of the area and mean fluorescence intensity).

### 2.8. Real-Time PCR

To assess viral NP gene expression, MDBK cells were infected with BCoV at MOI 0.5 and treated or not with 6PP for 48 h. Then, total RNA was extracted by Trizol reagent (Thermo Fisher, Waltham, MA, USA) and reverse transcribed into cDNA with an All-In-One RT MasterMix (Applied Biological Materials, Richmond, BC, Canada). The cDNA was amplified via real-time PCR using a BlasTaq 2 qPCR mastermix (Applied Biological Materials, Richmond, BC, Canada) and 0.1 μM primers from Eurofins. The primer sequences used were as follows: glyceraldehyde 3-phosphate dehydrogenase (GAPDH) forward (5′-CGGAGTCAACATTTGGTCGTAT-3′) and reverse (5′-AGCTTCTCCATGGTGGGGGGTGGTGAAGAC-3′); NP, forward (5′ GGACCCAAGTAGCGATGAG-3′) and reverse (5′-GACCTTCCTGAGCCTTCAATA-3′) [37,55]. The target gene expression was normalized to the housekeeping gene to determine the threshold cycle (Ct). Finally, gene expression levels were quantified by the 2−ΔΔCt method.

### 2.9. LysoRed Staining

In order to evaluate the effect of 6PP treatment on pH of lysosomes in MDBK cells during BCoV infection, Lysored staining was performed. Cells were infected or not with BCoV, at MOI of 0.05, and treated or not with 6PP (0.1 μg/mL) for 72 h, were then stained with a CytoPainter LysoRed Indicator Reagent (Abcam, Cambridge, UK), following the user manual [37,56]. After that, the cells were washed and analyzed by a microscopic ZOE Fluorescent Cell Imager (Bio-Rad Laboratories). The fluorescence intensity from microscopy images was obtained by ImageJ, as above. Statistical analysis was carried out on results from 3 images/group.

### 2.10. Three-Dimensional Structure Prediction and Molecular Docking Studies

Due to the lack of the high-resolution structure of bAhR in the PDB database, the three-dimensional (3D) model of the N-terminal region (residues 1–400) of the receptor, was predicted, as reported in a previous manuscript [20] using AlphaFold 3.0 (Figure S2) [57]. Briefly, the structural model was predicted with one homo-oligomer, MMseqs2 option for multiple sequence alignment (MSA) searching, in unpaired mode for generating separate MSA for each protein and no filter options for pair_cov (minimum coverage with query (%)) and pair_qid (minimum sequence identity with query (%)). The structural models were generated using the following setting parameters: number of models = 5; max recycles = 3. Therefore, we calculated five three-dimensional conformers showing a high degree of structural similarity on the level of secondary and tertiary structure organization and then the 3D structural model with the highest rank, based on pLDDT (predicted Local Distance Difference Test), was selected as reference structure and used, as reported below, in the molecular docking studies. The pLDDT is a per-residue measure of local confidence that assumes values within the range from 0 to 100, a higher value indicates high confidence and usually, a more accurate prediction.

The stereochemical quality of the representative model was assessed by evaluating the Ramachandran plot (Figure S3) obtained using the software PROCHECK v3.5 [58]. After that, the generated and validated 3D structural model of the receptor was used for molecular docking studies as described below. The 3D structure of the compound 6-pentyl-α-pyrone (6PP) was prepared using the 3D molecular tool of ChemDraw v. 19.0, and then a protocol for geometry optimization was applied. Molecular docking simulations were performed using AutoDock 4.0. The preparation of both the ligand and the receptor was performed using AutoDockTools, which included the addition of polar hydrogens, the Gasteiger charges, the torsional degrees of freedom for the ligand, and conversion to the PDBQT format. The docking grid was centered on the predicted ligand-binding region, with a cubic grid box of 40 × 40 × 40 with a spacing of 0.375 Å was centered in the ligand-binding region, encompassing the PASB domain. The genetic algorithm used was Lamarckian (LGA). Docking parameters included population size, a maximum number of energy assessments set to 2,500,000, and 100 independent runs. All resulting docking calculation poses were clustered using an RMSD cutoff of 2 Å. Molecular docking simulations resulted in five different clusters of binding poses. Among these, the most populated cluster and the conformation with the lowest binding energy were selected. The top-scoring pose was selected for interaction analysis. The structure of the complex was examined using PyMOL v. 3.1 [59] and Chimera v. 1.18 [60].

### 2.11. Statistical Analysis

Results are indicated as mean ± S.D. One-way ANOVA with Tukey’s post-test and by Student’s *t* test was assessed by GraphPad Prism 10.5.0 (GraphPad Software, San Diego, CA, USA). The n value represents the number of biological replicates for each figure. *p* < 0.05 was statistically significant.

## 3. Results

### 3.1. Determination of Cytotoxicity Time-Course

**Figure 2:**
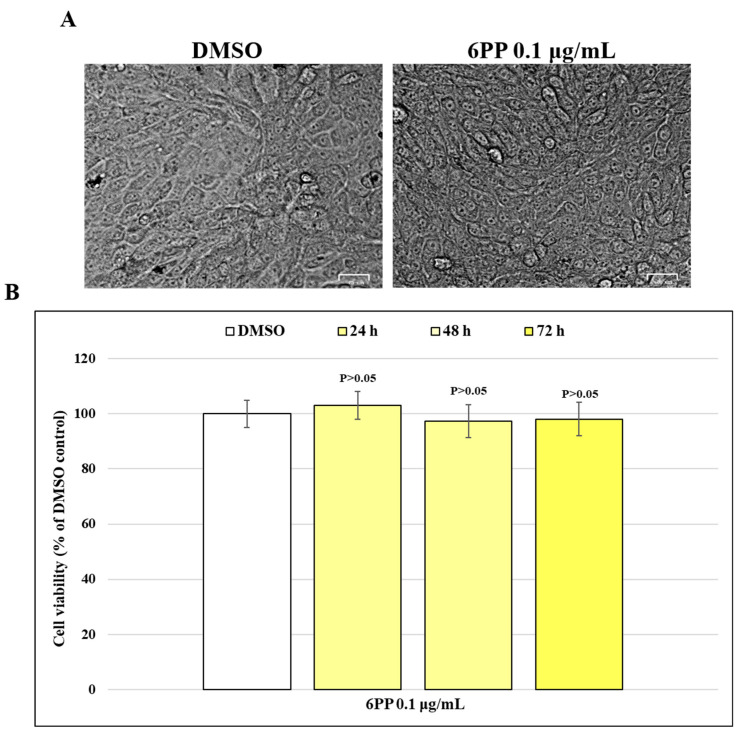
Cytotoxicity time-course of 6PP in MDBK cells. (**A**) MDBK cells were exposed at concentration of 0.1 µg/mL of 6PP and checked by a light microscope; the scale bar represents 25 µm. (**B**) Cell viability of MDBK cells treated with 6PP (0.1 µg/mL) by MTT test over the full 72 h experimental period. Significant differences between DMSO and 6PP-treated cells at different times of exposure are indicated by probability *p*. *p* > 0.05. Results show the mean ± S.D. of three independent experiments, n = 3.

Cytotoxicity time-course of 6PP (0.1 μg/mL) was assessed on MDBK cells for 72 h. After 72 h of treatment 6PP-treated cells were observed under a light microscope (Figure 2A). The MTT assay test was performed at 24, 48 and 72 h of treatment, reporting % of mortality or cell viability (% of DMSO control). Cytotoxicity time-course results demonstrated that cell viability is not compromised at the 0.1 μg/mL concentration over the full 72 h experimental period (Figure 2B).

### 3.2. Antiviral Activity of 6PP

**Figure 3:**
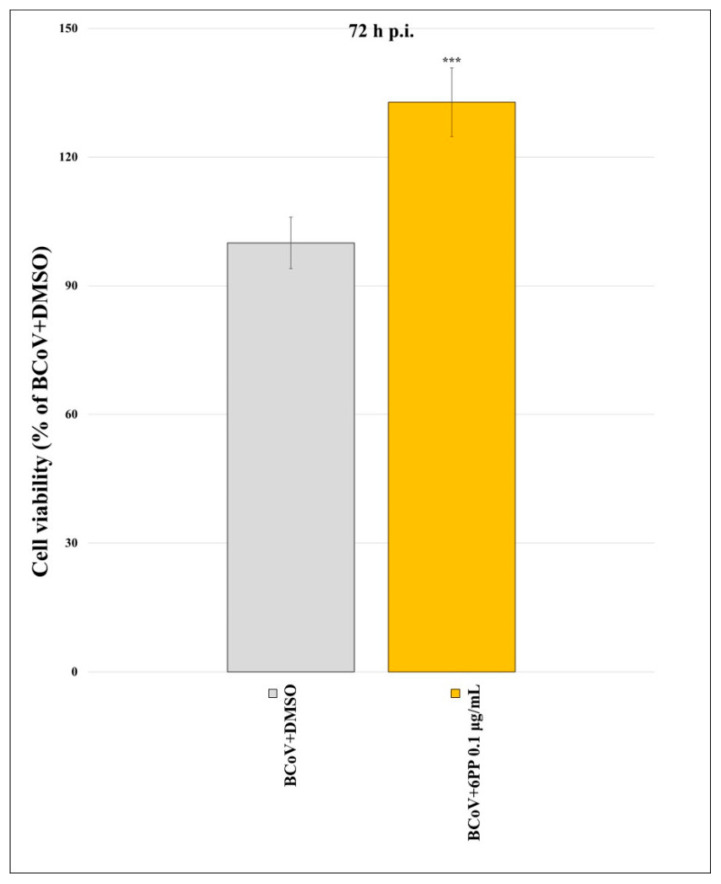
6PP-induced viral inhibition during BCoV infection. MDBK cells were infected with BCoV and treated or not with 6PP (0.1 µg/mL) for 72 h. MTT assay was performed and plotted values were calculated as the percentages relative to BCoV-treated cells with 6PP (BCoV+6PP) compared to BCoV-untreated cells (BCoV+DMSO). Significant differences between BCoV+DMSO and BCoV+6PP-treated cells are indicated by probability *p*. *** *p* < 0.001. Results show the mean ± S.D. of three independent experiments, n = 3.

An antiviral effect was obtained following cell viability during BCoV infection. Indeed, MDBK cells were infected with BCoV at MOI of 0.05 and treated with 6PP (0.1 µg/mL). After 72 h p.i., a significant (*p* < 0.001) viral inhibition during BCoV infection was observed in the presence of 0.1 µg/mL 6PP in infected cells, by performing the MTT assay (Figure 3).

**Figure 4:**
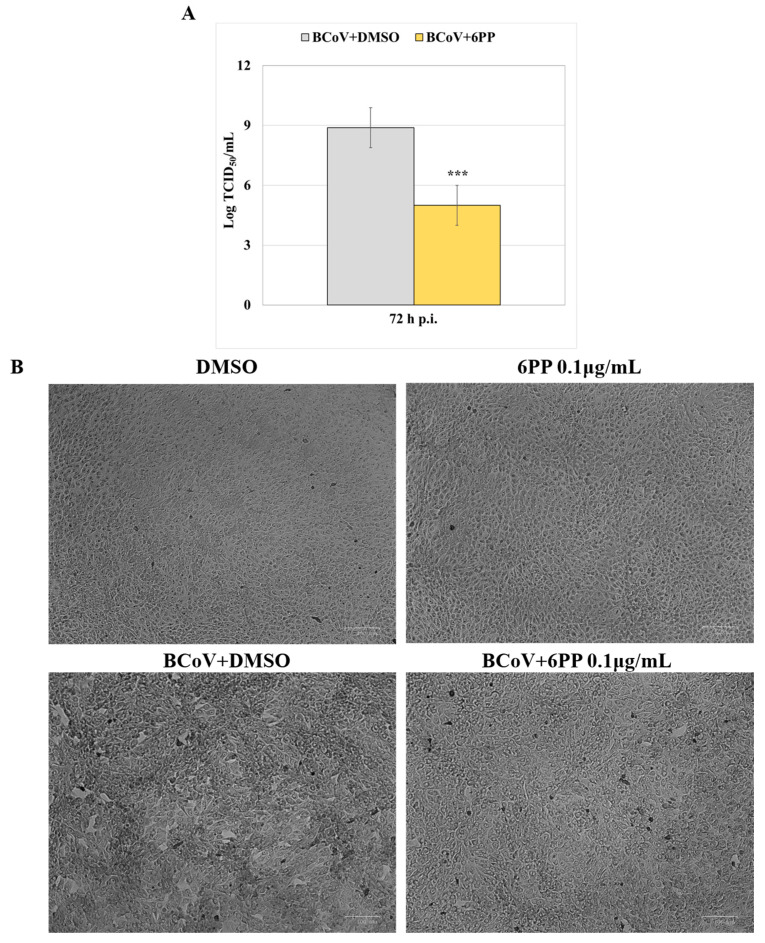
6PP decreased BCoV viral yield during infection in MDBK cells. Cells were infected or not with BCoV and treated or not with 6PP (0.1 µg/mL) for 72 h p.i. (**A**) Viral production was quantified using the TCID_50_ assay and expressed as Log TCID_50_/mL. Statistically significant differences between BCoV+DMSO and BCoV+6PP are indicated by probability *p*. *** *p* < 0.001. (**B**) The cytopathic effect (CPE) was evaluated using a ZOE Cell Imager. Scale bar 100 µm. Results are shown from one of three independent experiments, n = 3.

To better evaluate the protection role of 6PP in BCoV infection, virus yield at 72 h p.i. was assessed. The treatment with 6PP (0.1 µg/mL) during BCoV infection at MOI 0.5 in MDBK cells significantly affected viral production. Indeed, the virus titer (expressed in Log) at 72 h p.i. was markedly reduced (*p* < 0.001) in BCoV+6PP cells compared to the BCoV+DMSO control group (Figure 4A). In addition, microscopic examination, performed at 72 h of treatment, did not show notable differences in 6PP compared to DMSO groups (Figure 4B). Whereas, following infection with BCoV at MOI 0.05 for 72 h, the infected group (BCoV+DMSO) showed a greater cytopathic effect (CPE) in untreated cells compared to the group treated with 6PP (BCoV+6PP) (Figure 4B).

Overall, our findings showed that 6PP led to a significant decrease in BCoV production during infection in MDBK cells.

**Figure 5:**
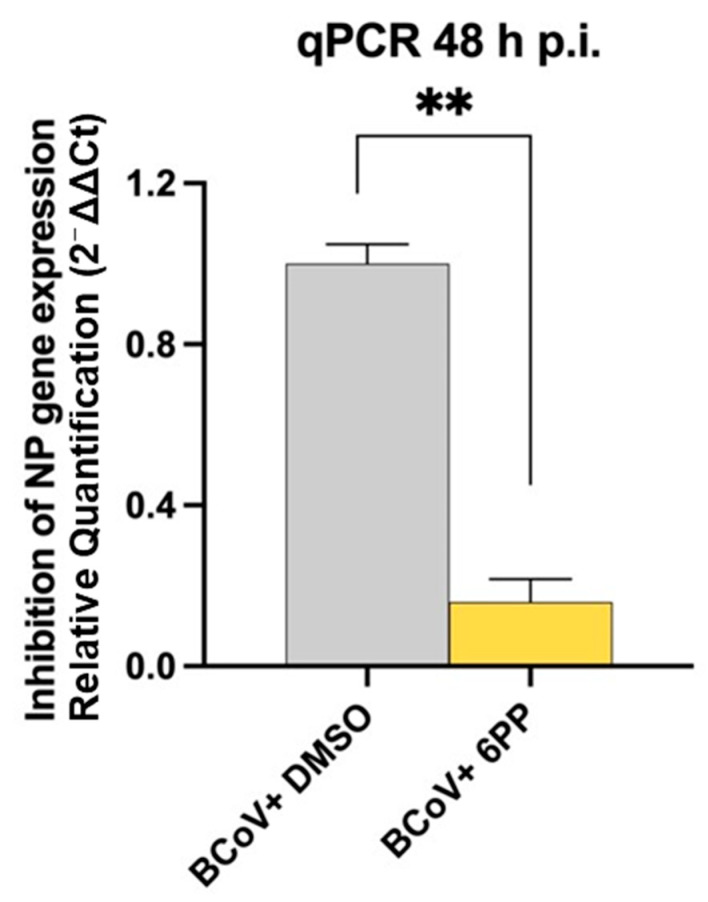
Treating MDBK cells with 6PP during BCoV Infection decreases NP gene expression. Error bars represent the standard deviations of the measurements. One-way ANOVA with Tukey’s Post Hoc Test was carried out. Significant differences in the *NP* gene expressions between the BCoV+DMSO and the BCoV+6PP groups are indicated; probabilities: *p*. ** *p* < 0.01. Results are shown from one of three independent experiments, n = 3.

The qPCR was employed to assess the ability of 6PP to suppress viral replication by measuring *NP* gene expression in MDBK cells infected with BCoV. Although the primer length was short, infected cells treated with 6PP showed a significant decrease in *NP* gene expression compared to untreated infected cells (Figure 5).

### 3.3. 6PP Reduces Signs of Cell Death Morphology During BCoV Infection in MDBK Cells

**Figure 6:**
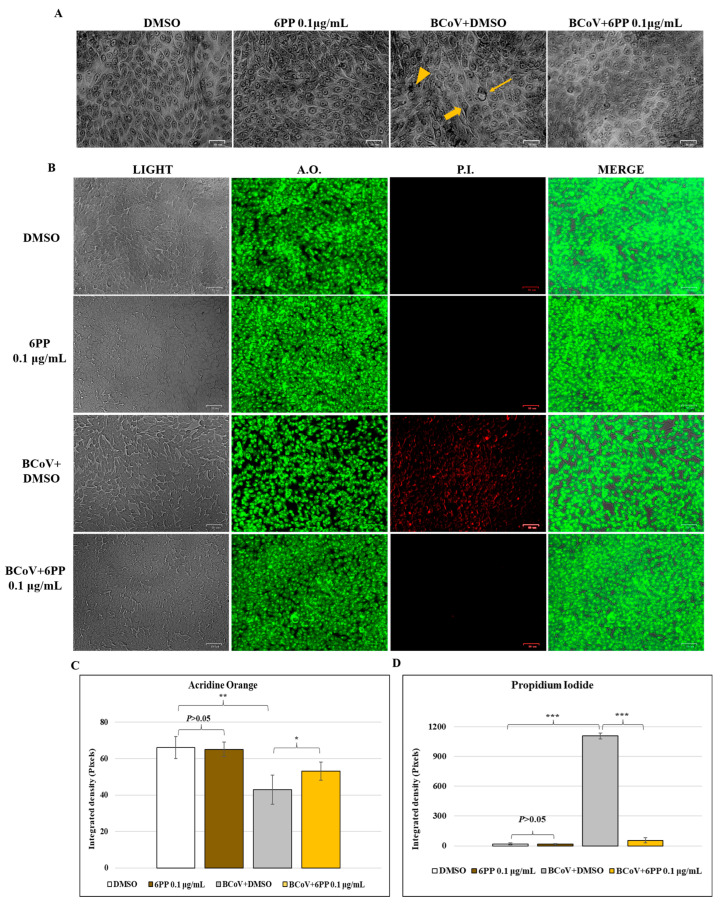

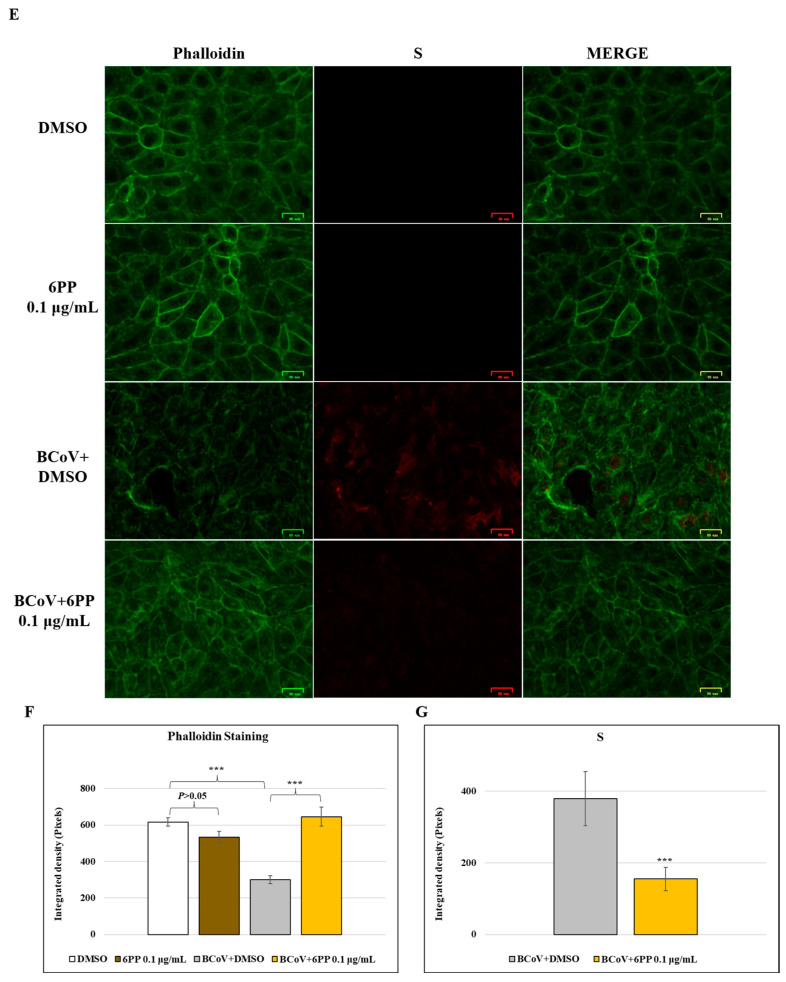
6PP reduced the morphological features of cell death and improved cytoskeletal rearrangements in MDBK cells during BCoV infection. Cells were treated or untreated with 6PP, infected or not with BCoV, and treated or untreated with 6PP (0.1 µg/mL) for 72 h. (**A**) Cells, after Giemsa staining, were examined under a light microscope. Morphological indicators of cell death, including cell shrinkage (arrow), nuclear pyknosis (arrowhead), and the presence of intercellular spaces due to cell detachment from the culture surface (slim arrow), were used. (**B**) In the AO/PI staining panels, PI fluorescent cells were identified. (**C**,**D**) Fluorescence density analysis of images displayed in (**C**). (**E**) Phalloidin-stained actin filaments of cell cytoskeleton (green), and anti-Bovine Coronavirus Spike antibody-stained S protein (red). (**F**,**G**) Fluorescence density analysis of images displayed in (**E**). Scale bar 25 µm and 50 µm. Significant differences between DMSO and 6PP, between DMSO and BCoV-infected cells, as well as between BCoV-infected cells and 6PP-treated infected cells are indicated by probability *p*. *** *p* < 0.001, ** *p* < 0.01 and * *p* < 0.05. The integrated density was assessed by ImageJ. Error bars represent standard deviation measurement. Results are shown from one of three independent experiments, n = 3.

Giemsa, AO/PI and Phalloidin staining were used to identify signs of cell death morphology. Following these different staining, no signs of morphological cell death in DMSO and 6PP groups were detected (Figure 6). Specifically, in untreated infected cells (BCoV+DMSO), cellular shrinkage (Figure 6A, arrowhead) and pyknosis (Figure 6A, arrow) were observed. These marks of cell death appeared reduced in BCoV-infected cells treated with 6PP (BCoV+6PP) compared to the untreated-infected group (BCoV+DMSO) (Figure 6A). Additionally, the increase in intercellular spaces due to cell detachment from the culture plate was primarily noted in untreated BCoV-infected cells (BCoV+DMSO) (slim arrow) when compared to the treated-infected group (BCoV+6PP) (Figure 6A). In the AO/PI panels, PI fluorescent cells, marking dead or dying cells, were predominantly found in BCoV-infected cells (BCoV+DMSO) rather than in 6PP-treated BCoV-infected cells (BCoV+6PP) (Figure 6B). In addition, in BCoV+6PP, the fluorescence density analysis (green) indicated a higher value than in BCoV+DMSO group (Figure 6C); conversely, a significant reduction in red signal was recorded in BCoV+6PP, indicating a protective role of 6PP during infection (Figure 6D). Phalloidin stained actin filaments of cell cytoskeleton in green, and anti-Bovine Coronavirus Spike antibody-stained S protein in red. In BCoV-infected cells (BCoV+DMSO), the specific marker for the presence of virus was detected in red, and a strong rearrangement in cytoskeleton organization, visualized by actin staining with Phalloidin, was detected in green (Figure 6E,F). In this group, there was a significant reduction in green fluorescence density (Figure 6E,F). In the presence of 6PP, we found an improved arrangement of cytoskeleton actin in BCoV-infected cells (BCoV+6PP), whose fluorescence density measure appeared intensely increased in green fluorescent signal (Figure 6E,F). In this group, a significant decline in red signal (S) was detected (Figure 6E,G).

These results demonstrated that 6PP provided significant protection to bovine cells during BCoV infection.

### 3.4. 6PP Causes a Reduction in the Expression of Cellular AhR Signaling During BCoV Infection

To clarify the role of 6PP on AhR signaling during BCoV infection at intermediate times, we investigated AhR and CYP1A1, its target protein, by immunofluorescence at 48 h p.i. In addition, the antiviral activity of 6PP was investigated by immunofluorescence, evaluating the expression of the viral S protein at 48 h p.i.

**Figure 7:**
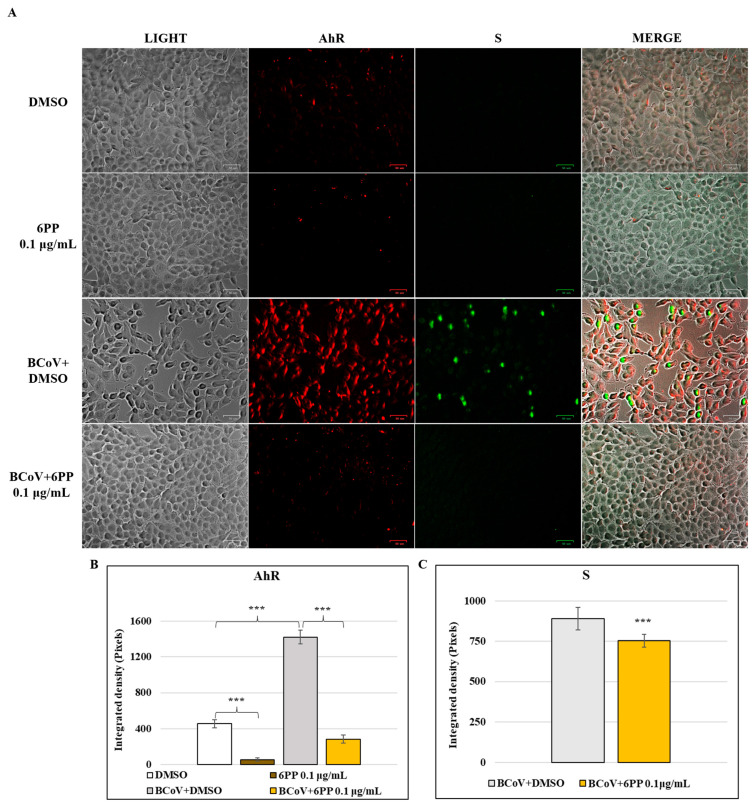
6PP induced a reduction in the expression levels of AhR and S proteins during BCoV infection in MDBK cells. MDBK cells were infected with BCoV at a MOI of 0.5 and either treated or left untreated with 6PP (0.1 µg/mL) for 48 h. (**A**) AhR and S proteins levels in both treated and untreated BCoV-infected groups were assessed by immunofluorescence. Scale bar 50 µm. (**B**,**C**). (**B**,**C**) Bar graphs show the statistical results of the mean ratio generated from the integrated density of the AhR and S proteins expression during BCoV infection for each group. Significant differences between DMSO and 6PP, between DMSO and BCoV-infected cells, as well as between BCoV-infected cells and 6PP-treated infected cells for AhR are indicated by probability *p*. *** *p* < 0.001. Significant differences between BCoV-infected cells and 6PP-treated infected cells for S protein levels are indicated by probability *p*. *** *p* < 0.001. The integrated density was assessed by ImageJ. Error bars represent standard deviation measurement. Results are shown from one of three independent experiments, n = 3.

**Figure 8:**
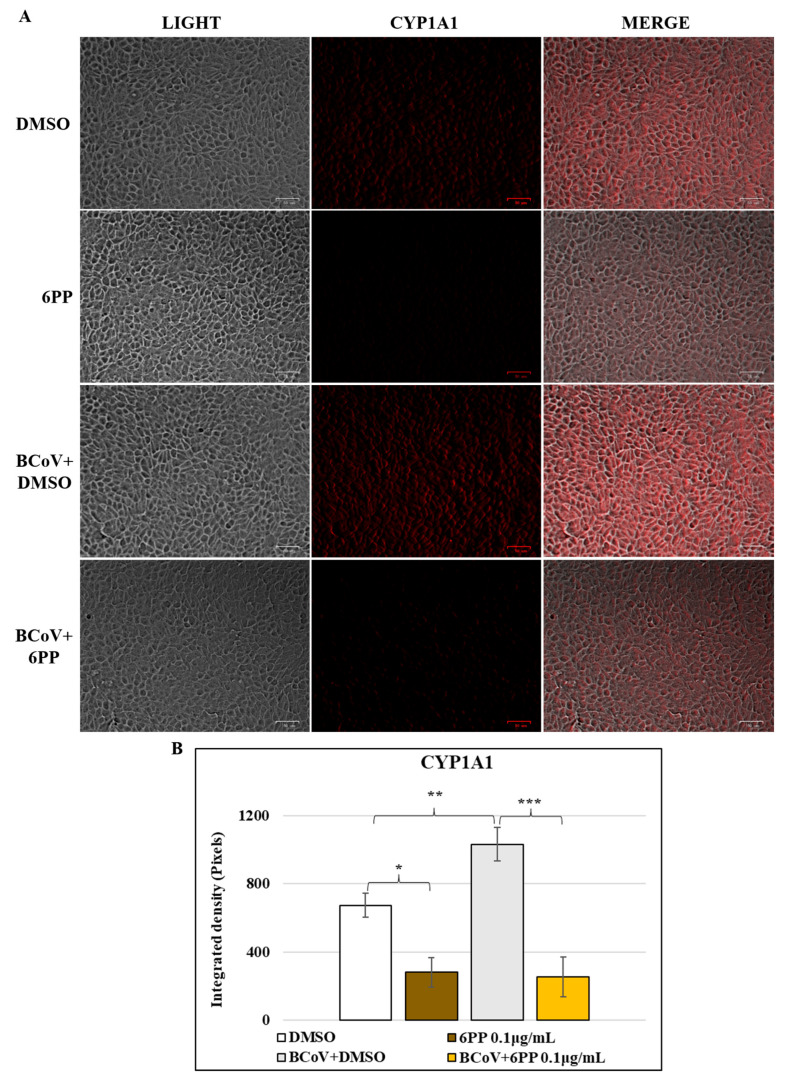
6PP provokes a downregulation in the expression of CYP1A1 during BCoV infection in MDBK cells. MDBK cells were infected with BCoV at an MOI of 0.05 and treated or not with 6PP for 48 h. Later, immunofluorescence for (**A**) CYP1A1 was carried out. Scale bar = 50 µm. (**B**) Bar graphs show the statistical results of the mean ratio generated from the integrated density of the CYP1A1 expression in MDBK cells treated with 6PP during BCoV infection for each group. Significant differences between DMSO and 6PP, between DMSO and BCoV infected cells, as well as between BCoV-infected cells and 6PP-treated infected cells for CYP1A1 protein, are indicated by probability *p*. *** *p* < 0.001, ** *p* < 0.01 and * *p* < 0.05. ImageJ was used to measure the integrated density. Error bars represent standard deviation measurement. The results are shown from one of three independent experiments, n = 3.

A reduction in the expression of cellular AhR protein was due to 6PP in MDBK cells, determined by comparing 6PP cells to DMSO control group (Figure 7A). 6PP compared to DMSO also caused a decline in the expression of CYP1A1 in MDBK cells (Figure 8A). During BCoV infection, a significant upregulation of AhR was observed in the BCoV+DMSO groups (Figure 7A), while the presence of 6PP induced a remarkable reduction (*p* < 0.001) of AhR in 6PP-treated infected cells (BCoV+6PP) (Figure 7A). An increased expression of CYP1A1 was found in BCoV-infected cells (Figure 8A), while treatment with 6PP provoked a significant downregulation of its expression in infected cells (Figure 8A). These results were confirmed by the analysis of integrated density (Figure 7B and Figure 8B).

Moreover, the expression of the viral S protein was assessed during BCoV infection in MDBK cells, and notably, the levels of viral S protein were markedly lower in infected groups treated with 6PP (BCoV+6PP) compared to untreated infected cells (BCoV+DMSO) (Figure 7A). These findings were confirmed by integrated density fluorescence quantification (Figure 7C).

### 3.5. 6PP Deacidifies Lysosomes During BCoV Infection in MDBK Cells

**Figure 9:**
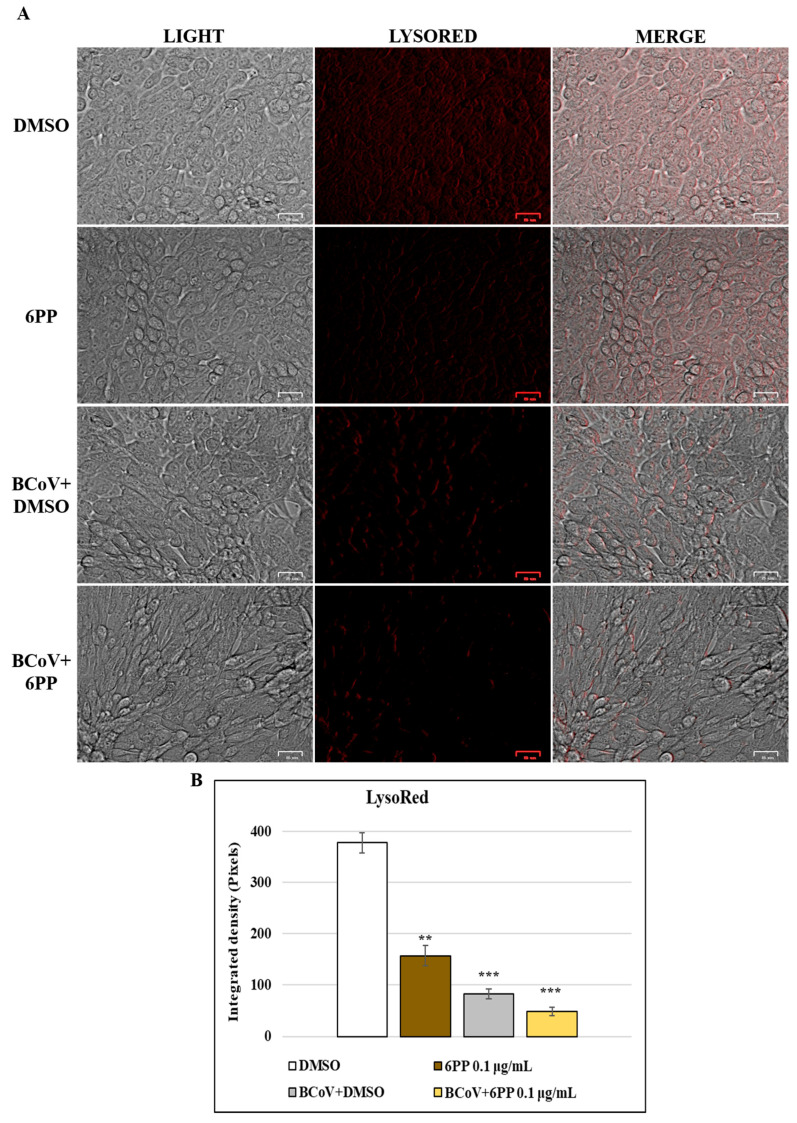
Lysosomes of MDBK cells were deacidified by 6PP during BCoV infection. MDBK cells were infected with BCoV at MOI of 0.05 and treated or not 6PP (0.1 µg/mL) (**A**). After 72 h, LysoRed of DMSO control group was compared to 6PP as well as to untreated BCoV-infected cells and 6PP-treated BCoV-infected groups. Scale bar 25 µm. (**B**) Bars indicate the mean ratio obtained by the integrated density of LysoRed obtained by ImageJ. Error bars indicate standard deviation quantification and significant differences are denoted by probability *p*. *** *p* < 0.001 and ** *p* < 0.01. The results are shown from one of three independent experiments, n = 3.

LysoRed, a red, fluorescent dye for identifying lysosomes in live cells, was employed to evaluate the acidity of the cellular organelles lysosomes during infection. Here, the presence of 6PP in MDBK cells provoked a marked deacidification of lysosomes (Figure 9A). During BCoV infection, the acidic environment of MDBK cell line resulted deacidified by BCoV infection (Figure 9A), as reported [20]. Treatment of BCoV-infected cells with 6PP (0.1 µg/mL) resulted in an additional deacidification of lysosomes during infection (Figure 9A). Integrated density analysis confirmed these results (Figure 9B).

### 3.6. Building the bAhR-6PP Complex by Molecular Docking

**Figure 10:**
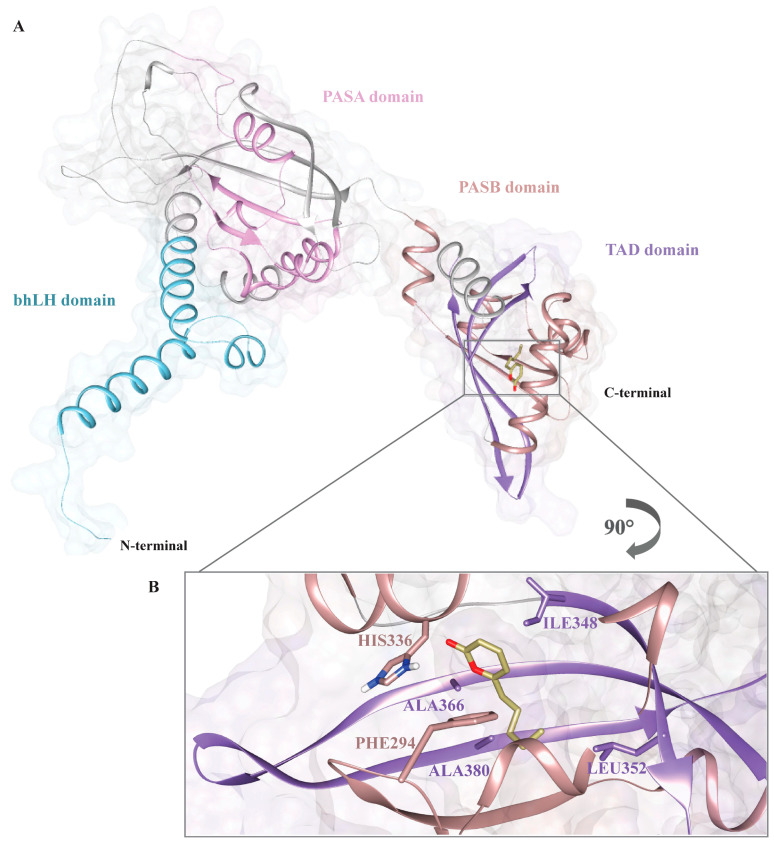
Structural representation of the protein–ligand complex highlighting key molecular interactions. (**A**) Schematic representation of AhR 1–400 structural domains: the bHLH domain (sky blue), PAS-A domain (plum), PAS-B domain (rosy brown), and TAD domain (purple). The N- and C-termini are indicated. (**B**) Close-up view of the ligand-binding site (dark khaki) located within the PAS-B domain. Key interacting residues are highlighted, including HIS336, PHE294, ILE348, LEU352, ALA366, and ALA380.

To examine the molecular determinants which drive the binding of bAhR by 6PP, we performed a sequence of molecular docking studies. After cluster analysis, we evaluated the stability of the indicative structural model of the bAhR-6PP complex, selected as reported in the Materials and Methods Section, by assessing its binding energy by the PRODIGY web server. The best-ranked pose of 6PP exhibited an estimated binding free energy of −4.80 kcal/mol. To note, the binding energy predicted for 6PP is similar to the value reported for the selective antagonist CH223191 in a previous study [20]. The docking 3D structural model revealed that 6PP fits into a binding pocket situated between the PASB and TAD domains of bAhR (Figure 10A). In particular, the ligand is stabilized only through hydrophobic contacts, including PHE294, HIS336, ILE348, LEU352, ALA366, and ALA380 (Figure 10B, Table S1). Yet, no significant hydrogen bonds or polar interactions were identified, demonstrating that the interaction is mainly driven by hydrophobic forces. The resulting binding profile revealed that 6PP presents binding interface similar to that observed for the selective chemical inhibitor CH223191 [20] (Figure S4). Taken together, the structural data, supported by in vitro assays results, indicate that 6PP, despite a slightly lower binding energy, presents a binding mechanism that is mainly mediated by a well-conserved hydrophobic cleft, as observed for CH223191 and other ligands [37]. These findings suggest that the 6PP ligand may be a good candidate for developing novel molecules able to modulate the receptor functional activities.

## 4. Discussion

Based on two different research projects aimed to investigate the potential antiviral activity of natural compounds against bovine coronavirus, in this study, during BCoV infection in MDBK cells, we investigated the action of 6PP, a fungal metabolite obtained by *Trichoderma atroviride*. The compound 6PP represents an interesting source for employing useful actions in various contexts, ranged from activity against plant pathogens [31,32] to anti-biofilm-producing bacteria [33]. Interestingly, 6PP has also shown in vitro antiviral activity against CCoV, an α-CoV [23]. In addition, in our previous paper, following BCoV infection with the strain 438/06, 6PP was used in different experimental conditions (e.g., temperature, pre- and p.i., and time of incubation) and a viral load lessening was detected in the presence of a modulation of viral entry [34].

Herein, with the aim of elucidating the potential activity of 6PP during BCoV infection, the involvement of AhR was investigated by in vitro and in silico studies. The co-treatment with no-toxic concentration (0.1 µg/mL) of 6PP resulted in a significant viral inhibition in MDBK cells infected with the strain 282/23 of BCoV. Particularly, during BCoV infection in MDBK, 6PP did not show a dose-dependent response curve. A similar trend was also obtained following BoHV-1 infection in MDBK cells by using another fungal SMs, like 3-O-methylfunicone, a benzo-γ-pyrone isolated by *Talaromyces pinophilus* [36]. In this study, 6PP has been shown to have anti-BCoV activity. Remarkably, similar results were found during infection with the same strain of BCoV in the presence of the specific AhR inhibitor CH223191, as well as of the fungal SM sphaeropsidin A (SphA) and its analog sphaeropsidin B (SphB) [20,37].

By using different cell-staining methods, marks of cell death morphology can be identified. Herein, Giemsa, as well as AO/PI staining showed morphological features of virus-induced cell death which resulted remarkably reduced in the presence of 6PP. A similar trend was also observed when SphA and SphB actively protect MDBK cells during BCoV infection [37]. Another simple method to detect cell death morphological signs can be obtained by using fluorescent Phalloidin, that showed the ability of BCoV for inducing morphological cell death features through actin reorganization. Generally, the complexity and dynamism of cytoskeletal network is due to protein filaments in the cytoplasmic environment of cells, whose main functions, related to cell morphology, contribute to cell resistance against mechanical alteration [60] and are implicated in different functions, both cell-dependent and virus-cell dependent, like viral phases of transport, assembly and release [61,62]. Particularly, during infection with another β-CoV, like SARS-CoV-2, in human pulmonary cells, intracellular rearrangements of actin were detected during viral assembly and release in the late phases of virus replication [57]. Here, BCoV caused an actin-dependent rearrangement, while a solid remodeling in cytoskeletal organization of actin in BCoV-infected cells was determined by 6PP, highlighting a protective role of this natural substance. The exact mechanism describing how 6PP interacts with cytoskeleton was not clear, but future studies may fully elucidate that modulation.

In addition, simultaneously, 6PP also determined a strong reduction in the virus yield, in the gene expression of viral N protein and in the expression of the viral S protein in virus-infected cells. The reduction in S protein signal may be a direct consequence of reduced viral replication induced by 6PP, rather than an inhibition of viral protein synthesis. Similarly, the reduction in *NP* gene RNA levels could not be interpreted as specific inhibition of *NP* RNA synthesis. Similar results were noticed by using different fungal SMs isolated by *Diplodia* species, like SphA and SphB [37].

Intriguingly, following 6PP treatment in BCoV infection, the modulation of AhR was evaluated, as it is known that the receptor modulates the immune response during infection by various viruses [11,14,15,16,18,20,21,23,24,26,37,63,64,65,66]. Herein, the natural increase in the expression of AhR signaling (AhR and its target protein CYP1A1) during BCoV infection [20,37] was confirmed, and fascinatingly, it was found to be significantly reduced by treatment with 6PP.

To promote their growth and replication, CoVs use the intracellular autophagic process [67]. β-CoVs enter cells, inducing lysosomal deacidification [38]. Indeed, BCoV non-structural protein 14 promotes viral replication and host immune evasion, by modulating the TNF receptor-associated factor 3 degradation, implicating the ubiquitin-proteasome as well as the autophagy-lysosomal signaling [68,69], processes which can be influenced by natural substances, including fungal SMs, that act as potential antivirals against CCoV [39], as well as BCoV [37]. Hence, the pH of lysosomes was evaluated during infection and the treatment with 6PP. Although the BCoV infection did not require an acidic environment, and fusion of BCoV viral membrane with a membrane of the host cell may be a low-pH-independent event [40], herein, following BCoV infection, a significant alkalinization of lysosomes was observed, probably due to cellular stress. This decrease in acidity was further enhanced in the infected groups treated with 6PP. Interestingly, a comparable deacidification of the lysosomal environment was observed in the presence of the fungal SMs SphA and B in MDBK cells infected with the same strain of BCoV [37].

In this study, molecular docking analysis has provided insights into the molecular determinants directing the recognition of the bAhR receptor by 6PP. The three-dimensional structural model obtained via molecular docking revealed that the ligand binds within the hydrophobic pocket positioned between the PASB and TAD domains of bAhR. In particular, the recognition mechanism of bAhR by 6PP is driven by an extensive hydrophobic network. Furthermore, comparison of the obtained 3D structure of the bAhR-6PP complex calculated for CH223191, the specific AhR chemical inhibitor [20], and natural compounds SphA and SphB [37], emphasizes that the receptor is featured by the presence of a preserved binding region.

Based on in vitro and in silico findings demonstrated herein, we think that studies are needed to clarify causal experiments (siRNA, knockout), as well as in vivo relevance. Future studies will focus on testing variants of the compounds and/or AhR to further validate the interaction profile. In addition, future studies will be designed to elucidate the action of 6PP on cytoskeleton as well as on cellular environmental acidity, which may reflect on virus–cell death.

## 5. Conclusions

In conclusion, our findings demonstrated that 6PP reduces BCoV replication in vitro and is associated with reduced AhR expression. Studying animal coronavirus infections with a One Health approach could lead to a translational study to examine the mechanisms of the SARS-CoV-2 infection in depth.

## Data Availability

The data that support the findings of this study are available from the corresponding authors upon reasonable request.

## Supplementary Materials

The following supporting information can be downloaded at https://www.mdpi.com/article/10.3390/pathogens15030332/s1, Figure S1: Gas Chromatography-Mass Spectrometry (GC-MS) analysis of purified 6-pentyl-α-pyrone (6PP) from Trichoderma atroviride strain P1. The main panel shows the Total Ion Chromatogram (TIC). The inset displays the corresponding electron ionization (EI) mass spectrum for this peak; Figure S2: Predicted three-dimensional structure of the N-terminal portion (residues 1–400) of the protein, generated using AlphaFold. The model includes the main structured domains: the basic Helix-Loop-Helix (bHLH) domain (sky blue), PAS-A domain (plum), PAS-B domain (rosy brown), and Transactivation (TAD) domain (purple). Domain boundaries are indicated in the top schematic and mapped onto the 3D structure. Only the N-terminal portion was modeled, as this region contains all known structured domains, while the rest of the protein (residues 401–848) is predicted to be largely disordered; Figure S3: The structural analysis of the three-dimensional model of the bovine Aryl Hydrocarbon Receptor (bAhR) was performed using an AlphaFold-generated model. The Ramachandran plot illustrates the distribution of dihedral angles, providing insight into the conformational properties and stereochemical quality of the predicted protein structure; Figure S4: Comparison of the bovine AhR binding pockets predicted for 6PP (a) and the reference antagonist CH223191(b). The three-dimensional structural models, obtained using same docking protocol, indicate that the two ligands, showing a comparable binding interface, are characterized, most likely, by a similar binding mechanism; Table S1: Representative table of the residues of the bAhR receptor involved in interactions with 6-pentyl-α-pyrone (6PP), as determined by docking analysis.
