# Supplementary material for: Potential Activity of 6-Pentyl-α-pyrone as an Antiviral for Bovine Coronavirus
Source: Pathogens. 2026 Mar 20;15(3):332. doi: 10.3390/pathogens15030332 (PMC13029080; doi:10.3390/pathogens15030332)
Supplement: Supplementary file 1 [file pathogens-15-00332-s001.zip › pathogens-4165066-supplementary.pdf]

pathogens

MDPI

Supplementary Materials

Potential activity of 6-Pentyl-α-Pyrone as an Antiviral for Bovine Coronavirus

Luca Del Sorbo[1,†], Rosa Giugliano[1,†], Clementina Acconcia[2,†], Maria Michela Salvatore[1], Alessia Staropoli[3,4], Violetta Iris Vasinioti[5], Maria Stella Lucente[5], Paolo Capozza[5], Francesco Vinale[1], Annamaria Pratelli[5], Luigi Russo[2], Rosa Iacovino[2,*] and Filomena Fiorito[1,*]

[1] Department of Veterinary Medicine and Animal Production, University of Naples Federico II, Naples, Italy; luca.delsorbo2@studenti.unina.it; rosa.giugliano@unicampania.it; mariamichela.salvatore@unina.it; frvinale@unina.it; filomena.fiorito@unina.it.

[2] Department of Environmental, Biological and Pharmaceutical Sciences and Technologies, University of Campania Luigi Vanvitelli, Caserta, Italy; clementina.acconcia@unicampania.it; luigi.russo2@unicampania.it; rosa.iacovino@unicampania.it.

[3] Department of Agricultural Sciences, University of Naples Federico II, Portici, Naples; alessia.staropoli@unina.it.

[4] Institute for Sustainable Plant Protection, National Research Council, Portici, Naples, Italy; alessia.staropoli@unina.it.

[5] Department of Veterinary Medicine, University of Bari, Valenzano, Bari, Italy; violetta.vasinioti@uniba.it; mariastella.lucente@uniba.it; paolo.capozza@uniba.it; annamaria.pratelli@uniba.it.

* rosa.iacovino@unicampania.it; filomena.fiorito@unina.it.

†These authors contributed equally to this work.

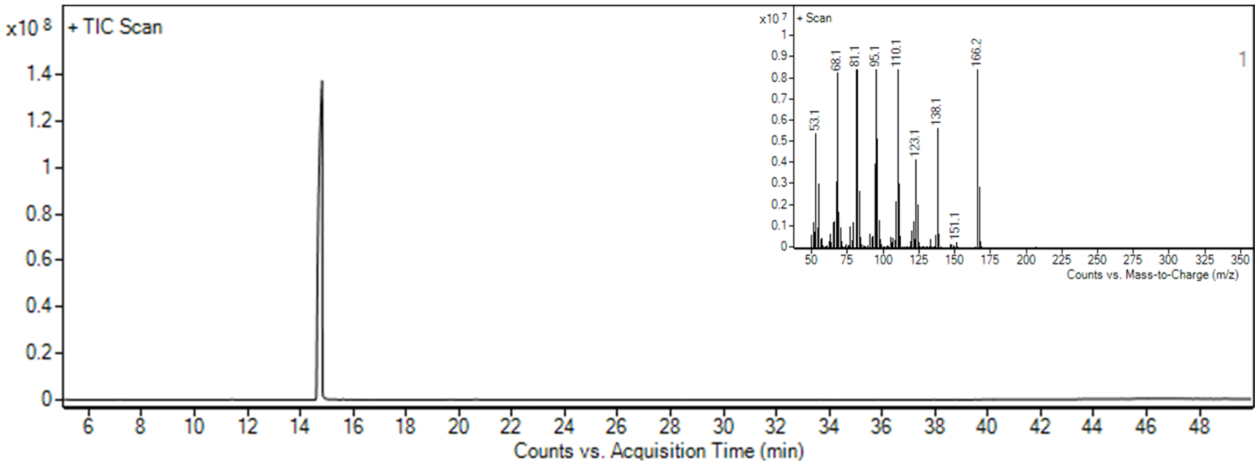

Figure SI1. Gas Chromatography-Mass Spectrometry (GC-MS) analysis of purified 6-pentyl-α-pyrone (6PP) from Trichoderma atroviride strain P1. The main panel shows the Total Ion Chromatogram (TIC). The inset displays the corresponding electron ionization (EI) mass spectrum for this peak.

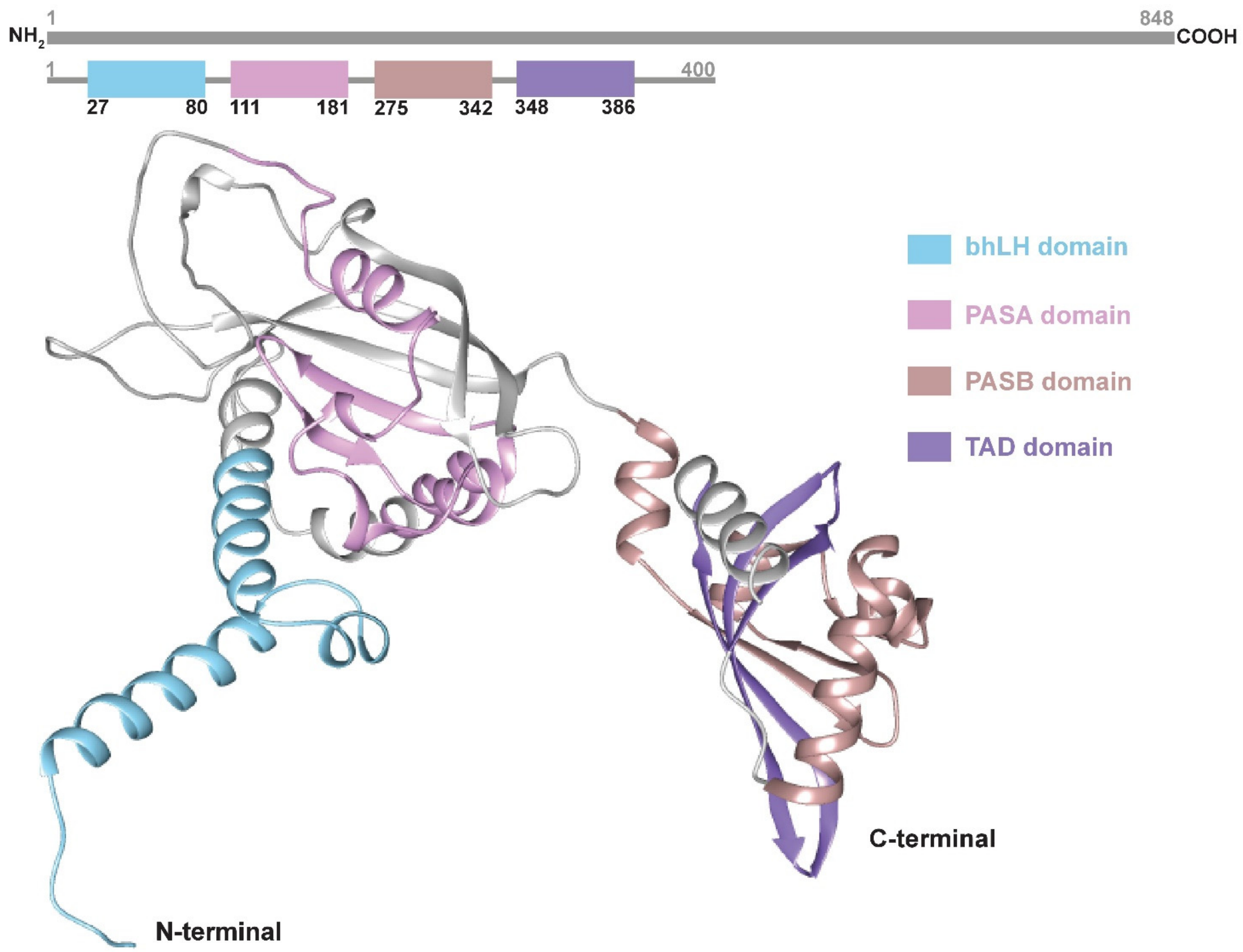

Figure SI2. Predicted three-dimensional structure of the N-terminal portion (residues 1–400) of the protein, generated using AlphaFold. The model includes the main structured domains: the basic Helix-Loop-Helix (bHLH) domain (sky blue), PAS-A domain (plum), PAS-B domain (rosy brown), and Transactivation (TAD) domain (purple). Domain boundaries are indicated in the top schematic and mapped onto the 3D structure. Only the N-terminal portion was modeled, as this region contains all known structured domains, while the rest of the protein (residues 401–848) is predicted to be largely disordered.

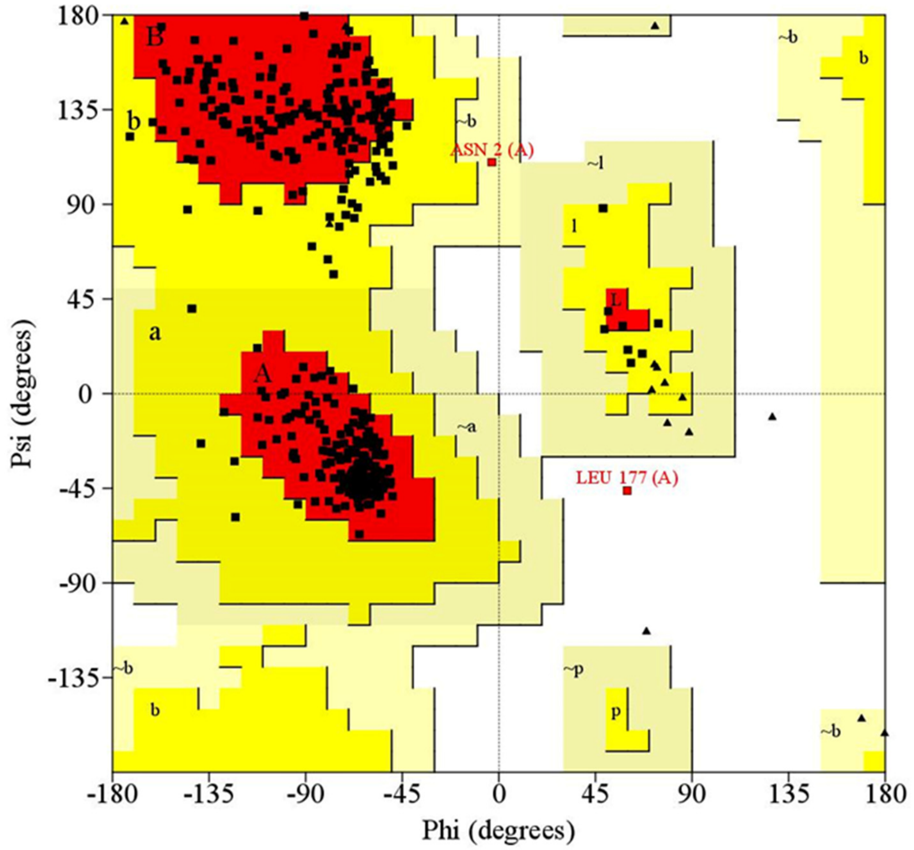

**Residues in most favoured regions**	**87.3 %**
**Residues in additional allowed regions**	**12.1%**
**Residues in generously allowed regions**	**0.3%**
**Residues in disallowed regions**	**0.3%**

Figure SI3. The structural analysis of the three-dimensional model of the bovine Aryl Hydrocarbon Receptor (bAhR) was performed using an AlphaFold-generated model. The Ramachandran plot illustrates the distribution of dihedral angles, providing insight into the conformational properties and stereochemical quality of the predicted protein structure.

Table SI1. Representative table of the residues of the bAhR receptor involved in interactions with 6-pentyl-α-pyrone (6PP), as determined by docking analysis.

	**HYDROPHOBIC INTERACTIONS**
1	PHE294
2	HIS336
3	ILE348
4	LEU352
5	ALA366
6	ALA380

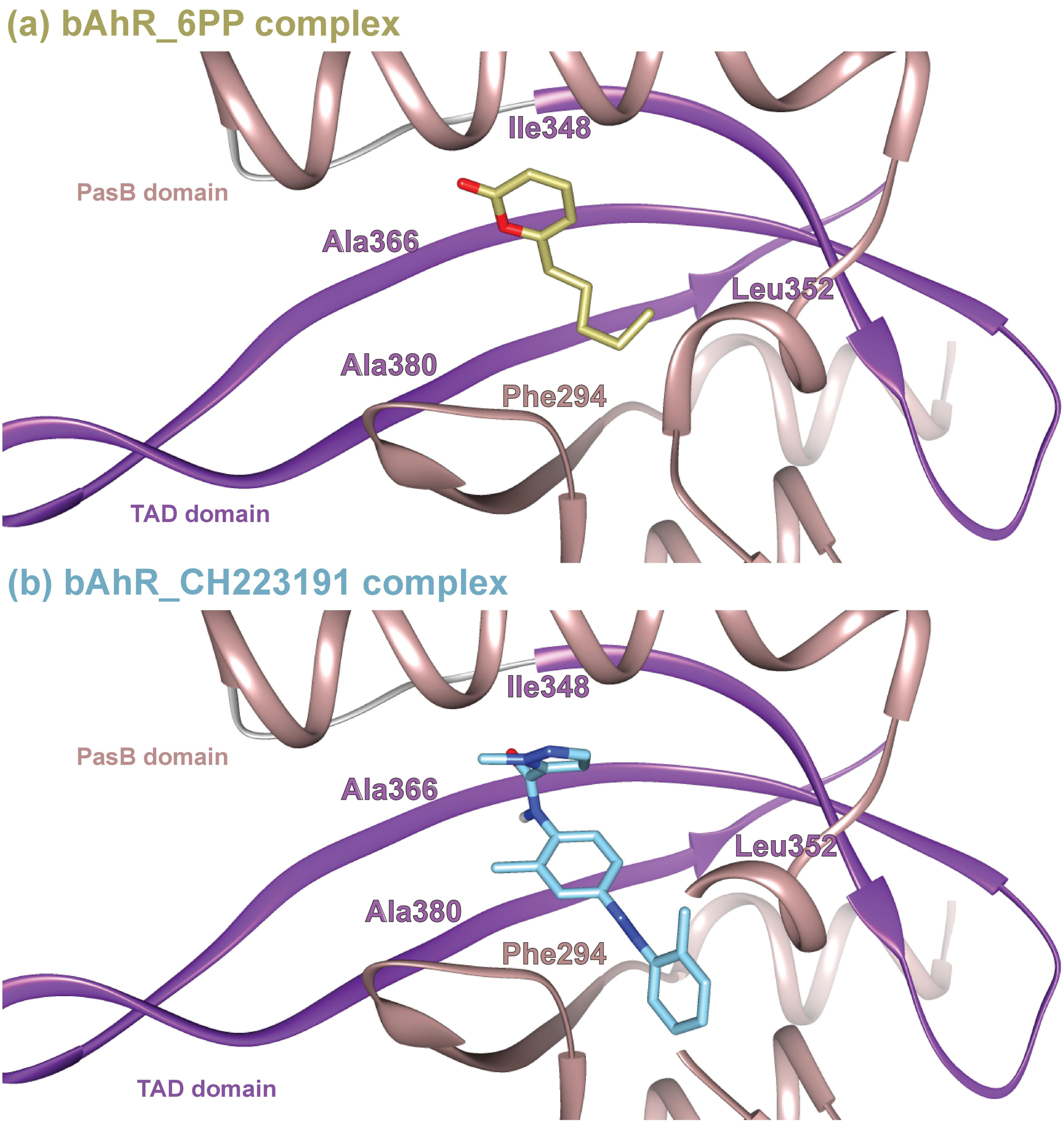

Figure SI4. Comparison of the bovine AhR binding pockets predicted for 6PP (a) and the reference antagonist CH223191(b). The three-dimensional structural models, obtained using same docking protocol, indicate that the two ligands, showing a comparable binding interface, are characterized, most likely, by a similar binding mechanism.
